# Congenital segmental dilatation of the jejunum in an African child: a case report

**DOI:** 10.11604/pamj.2021.38.122.27509

**Published:** 2021-02-03

**Authors:** Trésor Kibangula Kasanga, Florent Tshibwid Zeng, Stéphane Jampy-Biaya, Anatole Nyembwe Mbuyi, Sébastien Mbuyi-Musanzayi

**Affiliations:** 1 Department of Surgery, University Clinics of Lubumbashi, University of Lubumbashi, Lubumbashi, Democratic Republic of Congo,; 2 Service of Surgery, Medicare Hospital, Lubumbashi, Democratic Republic of Congo

**Keywords:** Congenital segmental dilatation, intestine, jejunum, children, case report

## Abstract

Congenital segmental dilatation of the intestine is a rare disease. It is rarely located in the jejunum and its etiology is still unknown despite many theories suggesting its mechanism. We report a case of a 17 months girl who experienced nonspecific symptoms (abdominal pain, constipation and loss of appetite) since early her infancy. She had no growth retardation and had moderate abdominal distension on physical examination. Investigations undertaken could not increase suspicion of congenital segmental dilatation of the intestine (CSDI). The diagnosis was made peroperatively and a resection was done, followed by end-to-end jejunal anastomosis. There were no postoperative complications and the patient is doing well after four months. One should think of CSDI in children with chronic subocclusion or digestive hemorrhage.

## Introduction

Congenital segmental dilatation of the intestine (CSDI) is a rare condition, characterized by localized increase of intestinal diameter. It was firstly described by Swenson and Rathauser in 1959 [1]. Since then, more than 150 cases have been reported worldwide [2]. The ileum is the most affected portion, while its jejunal location is rare. Most cases are diagnosed postnatally [2]. We report a case of a 17 months girl, admitted to our service for chronic abdominal pain and constipation, who has been peroperatively diagnosed a segmental dilatation of the jejunum. To our knowledge, it is the first ever reported case of CSDI in sub-Saharan Africa. We discuss different aspects of CSDI in general (location, criteria, etiology) and those of its jejunal location in particular (antenatal diagnosis, symptoms, investigations, management, microscopic features and outcomes).

## Patient and observation

A 17 months girl has been consulted in our service for colicky abdominal pain often associated with constipation and loss of appetite, since she was six months old. Parents kept her at home until they remarked visible peristalsis on the abdominal wall, which lead them to consult our service. She was born from a full-term and uncomplicated pregnancy during which the mother had no prenatal ultrasound (US). Her neonatal period was unremarkable.

**Clinical findings:** on physical examination, she was colored and active. Her vitals were within normal limits as well as her anthropometric parameters (weight: 10kg). Her abdomen had moderate distension and peristaltic waves were visible on the abdominal wall. Abdomen was soft on palpation and tympanic on percussion. The digital rectal examination was unremarkable. Symptomatic treatment was initiated with lactulose (five mL bid) and an antispasmodic, trimebutine (five mL bid).

**Figure 1:**
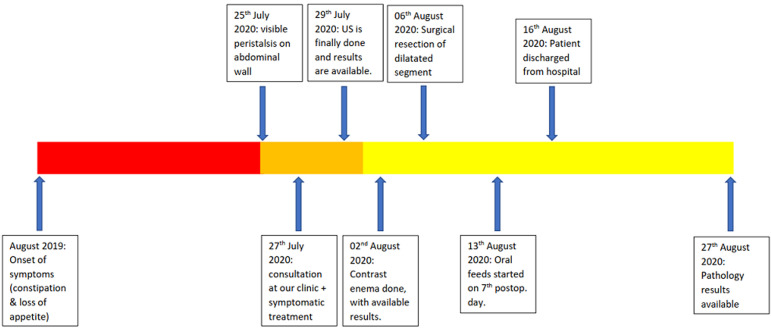
timeline showing historical and current information

**Timeline:** our patient experienced first symptoms since August 2019. Parents brought her at our service only on 27^th^ July 2020 two days after they remarked visible peristalsis on her abdominal wall. The patient has been treated and discharged in August 2020 and pathology results were available 11 days later (Figure 1).

**Figure 2:**
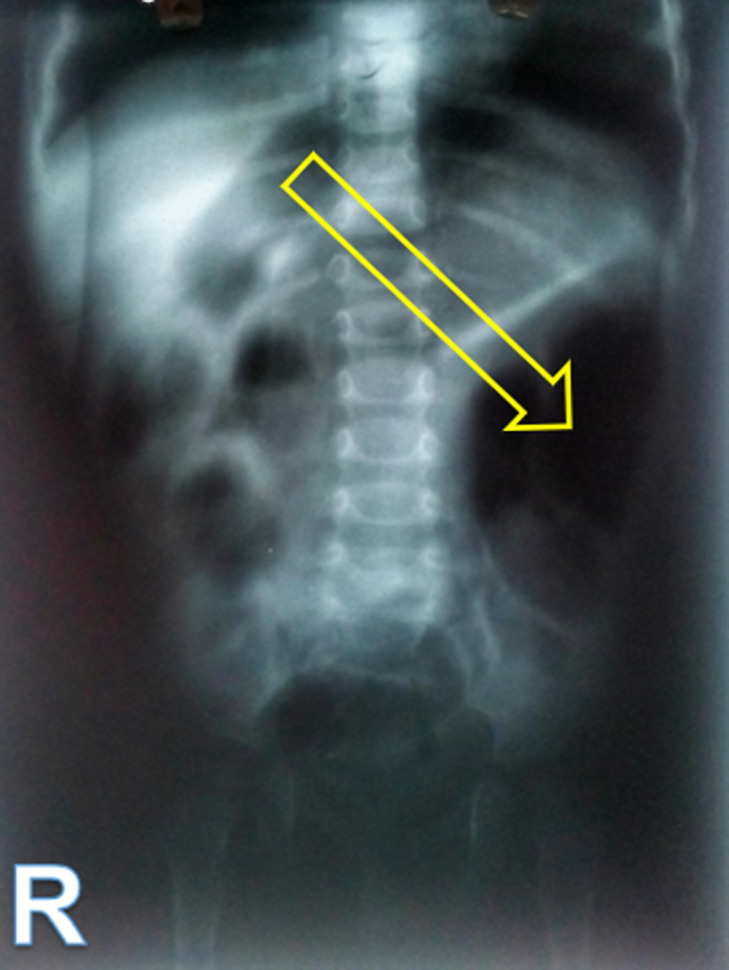
plain abdominal X-ray showing a distended intestinal loop without air-fluid level in the left lumbar region (yellow arrow)

**Diagnostic assessment:** blood results were within normal range. Abdominal X-ray showed distension of an intestinal loop in the left lumbar region (Figure 2). Abdominal US identified a misleading colonic dilatation containing heterogeny echoic substance, with no other additional features. Contrast enema was unremarkable. Due to financial constraints, upper gastro-intestinal (GI) series and abdominal computed tomography (CT) could not be performed. An exploratory laparotomy has been indicated with presumptive diagnosis of intestinal duplication.

**Figure 3:**
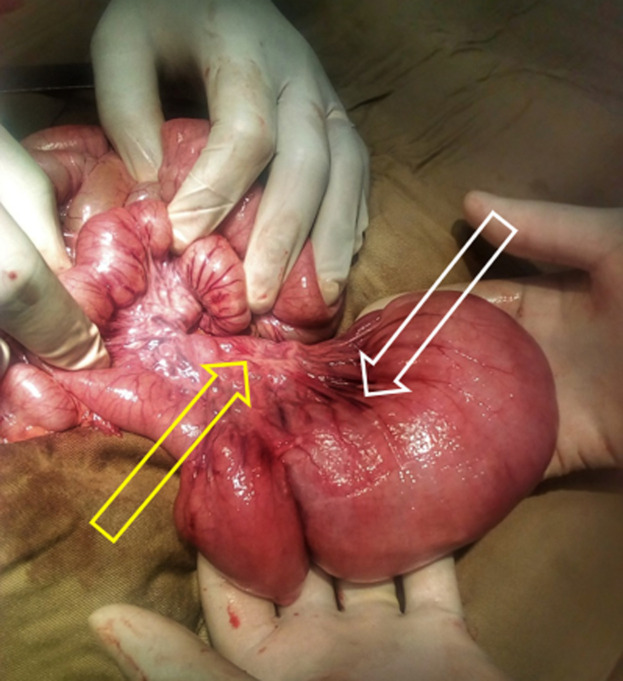
intraoperative findings showing the dilatated jejunum. Note prominence of serosal vessels (white arrow) and thickening of the adjacent mesentery (yellow arrow)

**Therapeutic intervention:** after the median laparotomy, a dilatated segment of the jejunum was identified at approximately ten cm from the Treitz´s angle. Its diameter was fourfold greater than of the adjacent bowel, to which the dilatation was connected abruptly, without any identified obstructive cause. It measured ten cm in length and had prominent serosal vessels. Adjacent mesentery was thickened (Figure 3). No further anatomic anomaly was identified. A resection of the dilatated portion was performed, followed by end-to-end jejunal anastomosis using Vicryl 3/0 in separate stitches.

**Follow-up and outcomes:** microscopic examination revealed a lymphocytic infiltration of both layers of the muscularis propria. The dense lymphocytic infiltration was mainly constituted of T lymphocytes CD3+/8+ (C and D) and some B lymphocytes CD20+. Locally diminished actin coloration indicated atrophy of smooth muscle fibers. Nerve fibers and ganglion cells of myenteric and submucosal plexuses were intact with focalized absence of NK CD56+ cells around lymphocytic infiltration. No ectopic tissue was identified. The postoperative period was uneventful. The patient was on parenteral nutrition until day seven postoperatively. She was discharged on day ten and after three months of monthly follow-up, colicky abdominal pain and chronic constipation had disappeared since the early postoperative days. For the future, the follow-up will be done on a three months basis for a year and then, twice yearly.

## Discussion

In this patient, strengths of our approach were to quickly rollout a colonic cause of constipation by doing a contrast enema. We decided to explore the patient instead of insisting to have a CT, which would certainly delay the treatment. We practiced the recommended treatment by resecting the dilatated segment. The main limitation was that CSDI was an intra-operative surprise, which could be avoided by obtaining upper GI series. In 1959, Swenson and Rathauser reported for the first time three cases of CSDI interesting the colon [1]. Since then, many other cases have been reported with the ileum being the most affected region (50 to 61.1% of cases), followed by the colon (26.4 to 35.7%), jejunum (9.7 to 10.7%) and rarely duodenum (2.8 to 3.6%) [2]. The first report of jejunal location have been published in 1973 by Rossi and Giacomoni [3]. Criteria of diagnosis of CSDI are: (a) localized intestinal dilatation with at least a threefold increase in diameter, (b) abrupt connection to adjacent normal bowel, (c) no cause of obstruction distal to the dilatation, (d) symptoms and signs of bowel subocclusion or occlusion, (e) presence of normal ganglion cells and nerve fibers, and (f) completely cured after surgical resection of the dilatated segment [2]. Etiology of CSDI is still not known despite more reported cases and advances in investigations. Several theories have been proposed to understand the pathogeny of this condition. Some of them suggested a vascular origin: abnormal tortuous vessels or intussusception leading to vascular insufficiency. Other suggested mechanical causes: kinking and volvulus in the first trimester of development, omphalocele sac ensnaring the bowel or strangulation in the umbilical ring in the early stages of development. Anomaly of the separation of notochord from the endoderm or ectopic tissue disturbing continuity of nerve fibers and intestinal plexuses have also been cited [2].

Antenatal diagnosis is possible by prenatal US or magnetic resonance imaging (MRI). Several reports described antenatal lesions appearing as a sonolucent mass changing during examination [4-6]. Patients with jejunal congenital segmental dilatation (CSD) present symptoms as early as the third hour of life or much more later as nine years old [4,7]. Symptoms include bilious vomiting and abdominal distension, constipation, abdominal pain, diarrhea and loss of appetite [4-8]. Patient are first investigated with plain abdominal X-ray, which usually shows distended intestinal loop associated or not to hydroaeric level [5-7]. Other investigations are upper GI series or contrast enema. The first allow diagnosis by showing a localized dilatation of the small intestine [4,6,8]. It also allows to identify anomalies of rotation and fixation (nonrotation, mobile cecum) [3]. Contrast enema helps to exclude colonic causes of constipation. Abdominal US is not of great help and shows nonspecific sign as bowel distention. In toddler, it helps to exclude an atypic presentation of intussusception. For jejunal location, surgical treatment consists of resection of the dilatated segment, followed by an end-to-end anastomosis with or without stoma [2]. Microscopic examination of specimen usually shows alteration of the muscularis propria, varying from atrophy to hypertrophy, vacuolization or inflammation [3-5,7,9]. In opposition to the ileal location, no ectopic tissue has ever been reported in jejunal CSD. Jejunal CSD has excellent outcome. Some authors reported unremarkable long-term follow-up lasting up to 10 years postoperatively [10]. The lesson to learn from this case is that children with chronic abdominal symptoms should be well investigated despite lack of equipment or financial constraints. If no current etiology of these symptoms is found, the clinician should think of rarer causes, such as CSDI which treatment is simple and efficient. This will make our patient to less suffer.

**Patient perspective:** parents of our patient were happy and unburdened that symptoms of their child regressed quite rapidly after the surgical intervention, especially constipation and visible peristalsis on the abdominal wall. They wish to have brought their child quite earlier, during the infancy when symptoms started.

**Informed consent:** the authors declare that a written consent for publication has been obtained from the father of the patient.

## Conclusion

Congenital segmental dilatation of the intestine is a rare condition and its jejunal location is rarer. One should also think about it when having children with nonspecific symptoms like vomiting and abdominal distention, especially if they started during or nearly after the neonatal period. Upper GI series gives the diagnosis and surgery is the cornerstone of treatment.
